# The Morphology of Nasal Polyps in Different Age Groups: Histopathological Features

**DOI:** 10.3390/jpm14040414

**Published:** 2024-04-14

**Authors:** Vincenzo Fiorentino, Maurizio Martini, Cosimo Galletti, Cristina Pizzimenti, Mariausilia Franchina, Antonio Ieni, Guido Fadda, Bruno Galletti, Giovanni Tuccari

**Affiliations:** 1 Dipartimento di Patologia Umana Dell’adulto e Dell’età Evolutiva Gaetano Barresi, Università Degli Studi di Messina, 98122 Messina, Italy; maurizio.martini@unime.it (M.M.); cosimogalletti92@gmail.com (C.G.); mariausiliafranchina@hotmail.it (M.F.); antonio.ieni@unime.it (A.I.); guido.fadda@unime.it (G.F.); bruno.galletti@unime.it (B.G.); giovanni.tuccari@unime.it (G.T.); 2 Dipartimento di Scienze Biomediche, Odontoiatriche e Delle Immagini Morfologiche e Funzionali, Università Degli Studi di Messina, 98122 Messina, Italy; cristina.pizzimenti@unime.it

**Keywords:** nasal polyps, inflammatory infiltrate, age-related morphology, biological therapeutical agents

## Abstract

Background: Nasal polyps (NPs) represent the end-stage manifestation of chronic rhinosinusitis (CRS), a relatively common pathological condition encountered in all ages of life. Methodology: The aim of our study was to evaluate the histological features and inflammatory cellular components of NPs in a retrospective cohort (143 cases) of pediatric, adult and elderly populations in order to discuss the possible morphological age-related differences statistically documented. Results: In the pediatric group, the inflammatory infiltrate presented many eosinophils mixed with lymphocytes, while in the adult population, lymphocytes and plasma cells were mainly evident, frequently with a perivascular distribution or with the formation of subepithelial lymphoid nodules. In the elderly population, inflammation was less evident and was associated with cavernous-like angecthatic structures with thrombotic stratification. Nearly all morphological findings exhibited statistically significant values among differently aged subgroups. Conclusions: Our results support the presence of histological specificities of NPs at different ages of life, providing new insight into the etiopathogenesis of NPs. The future role of biological therapies, mainly in cases refractory to already available standard medical and surgical treatments, may be analyzed by a prospective study using a larger cohort with a long-term evaluation also in relation to a possible relapse.

## 1. Introduction

Nasal polyps (NPs) represent the end-stage local manifestation of a chronic inflammatory disease affecting the nasal cavity and paranasal sinuses [1]. Although they are mainly related to chronic rhinosinusitis (CRS), they can also be associated with aspirin-exacerbated respiratory disease, systemic vasculitis, and cystic fibrosis [1]. According to European guidelines, the inflammatory condition underlying NPs affecting the nose and paranasal sinuses should be associated with two or more symptoms, with at least one of them being nasal blockage, obstruction, or congestion, as well as nasal discharge, facial pain or pressure, and reduction or loss of smell [1]. Furthermore, the presence of mucopurulent discharge, as well as edema and mucosal obstruction, primarily in the middle meatus, may strongly support the diagnosis [1]; via imaging, mucosal changes within the osteomeatal complex and/or sinuses have been revealed through the use of computed tomography (CT) [1].

Morphologically, NPs grossly appear as edematous grape-like protrusions that typically arise in the upper nasal region, specifically next to the osteomeatal complex situated on the lateral wall [2]. Antrochoanal polyps are typically found in children, where they represent up to 42% of all NPs, while they are less frequent in adults, where they represent only 4–6% of all NPs [3,4,5]. On the other side, in the adult population, the majority of NPs arise from the ethmoidal sinuses [2]. The surface epithelium of NPs is typically characterized by its smooth texture and composition of pale translucent tissue, thereby setting it apart from the highly vascularized mucosa found within the nasal cavity [2]. NPs are frequently bilateral and can vary in size from a few millimeters to several centimeters [2,3,4,5]. Furthermore, data taken from the literature show that they are more frequent in older age groups [6,7] and in men [6], with an average age at diagnosis of 40 to 60 years old [8] and a prevalence of 5% in individuals aged 60 years old or older [6]. However, other authors have reported a greater incidence of such a pathology in women aged 51–60 years old with clinical symptoms of nasal obstruction [9]. On the other hand, NPs are reported as being rare in children and young people [10], even if they can affect up to 50% of cystic fibrosis patients [11]. Therefore, these findings suggest that age may play a role in the development of such a pathology.

NPs originate from mucosal epithelial rupture, fibrous tissue proliferation, extracellular matrix accumulation, and inflammatory cell infiltration [12]. In fact, histologically, they are characterized by an infiltration of inflammatory cells, including eosinophils, neutrophils, mast cells, plasma cells, lymphocytes, and monocytes associated with structural fibrosis, edematous stromal tissue, and basement membrane thickening [13,14,15,16]. Interestingly, the type of predominant inflammatory cells has been linked to their different clinical features [17]. In fact, the spectrum of inflammatory patterns in CRS includes eosinophilic, neutrophilic, and mixed inflammatory patterns [17]. The presence of high levels of eosinophils has been related to the perpetuation of the inflammatory process in CRS with nasal polyps (CRSwNPs) [18]. Moreover, eosinophilic inflammation has been associated with more severe disease, higher rates of recurrence after surgery, and a greater likelihood of comorbid asthma and allergies [19,20]. Additionally, it has been shown that NPs with strong eosinophilic infiltration are more likely to recur after endoscopic sinus surgery [19,20]. This latter evidence suggests that the presence of eosinophils may be associated with more aggressive clinical behavior and poorer treatment response in such diseases [20]. On the other hand, neutrophilic inflammation has been linked to less severe disease, a lower likelihood of comorbid asthma and allergies, and a better response to antibiotics [17].

Only a few reports have analyzed the histological differences of NPs regarding both their morphological features and the different cellular components of inflammatory infiltrates found in different age groups [2,5]. In detail, an allergic pattern (rich in eosinophils) in histology was more common in the pediatric population, while an inflammatory pattern (rich in neutrophils) predominated in the adult population [2]; interestingly, this difference was found to be statistically significant (*p* value < 0.01) [2]. In addition, allergic polyps were significantly more common than inflammatory ones among children, while in adults, inflammatory polyps were more common as elsewhere reported [5].

In light of these few observations concerning NPs’ age-related characteristics, we though that an investigation of a cohort of such lesions would be of interest in terms of verifying whether the morphological characteristics change between pediatric, adult, and elderly age groups. Therefore, we aimed to examine the qualitative histological features as well as the composition of the inflammatory infiltrate observed in the NPs of patients of different ages, also verifying if these aspects may acquire a statistically significant value.

## 2. Materials and Methods

We have retrospectively analyzed NPs from 143 patients who underwent functional endoscopic sinus surgery (FESS) at AOU Policlinico G. Martino, taken from files of pathological archives concerning the period March 2013–August 2023. Firstly, the study population was subdivided into pediatric (≤18 years of age), adult age (between 18 and 50 years old), and elderly age (>50 years old) groups for statistical purposes. The reason for such an age subgrouping finds its explanation in the literature data that show how, in over 50 year olds, the nasal cycle appears to be significantly reduced [21], and the prevalence of both CRS [22] and NPs sharply increases after the age of 50 years [8,23]. As proof of this, many studies regarding CRSwNPs have used 50 years as the cut-off age for the elderly patient subgroup [22,24,25,26].

The only inclusion criterion for participation in this study consisted of a clinical and radiological diagnosis of NPs with subsequent surgical excision. All of the patients in our study suspended their therapies (topical and/or systemic corticosteroid burst and taper dosages, as well as leukotriene receptor antagonists) at least 4 weeks before surgery, so the impact of treatment on histopathology can be considered negligible.

On the other side, our study excluded patients from whom there was no pathological specimen, patients who had received topical and/or systemic corticosteroid burst and taper dosages as well as leukotriene receptor antagonists within 4 weeks before surgery and those whose information on such treatments was not available. Moreover, patients with upper respiratory disease other than CRSwNP, which also included asthma and allergies, such as sensitivity to acetylsalicylic acid or other non-steroidal anti-inflammatory drugs (NSAIDs), were excluded. Other exclusion criteria comprised autoimmune disorders, infections, and malignancies.

Ethical review and approval were waived for this study due to its retrospective nature. All patient data were collected anonymously, and written informed consent, as part of the routine diagnosis and treatment procedures, was obtained from patients or their guardians in accordance with Good Clinical Practice guidelines and the Declaration of Helsinki (1975, revised in 2013). The clinical information was retrieved from the patients’ medical records and pathology reports. Patients’ initials or other personal identifiers do not appear in any image.

All of the histological specimens were fixed using 10% buffered formalin, with an exposure duration ranging from 12 to 48 h at room temperature. Subsequently, paraffin-embedded tissue blocks were obtained from which 4–5 µm-thick sections were cut and routinely stained using hematoxylin and eosin (H&E). At least two independent and well-experienced pathologists retrospectively examined H&E-stained slides of NPs to analyze the nature of the inflammatory infiltrate (defined as the number of inflammatory cells per 10 high power fields (HPF)) and their morphological qualitative features (the presence of lymphoid nodules, squamous metaplasia, and vascular ectasias). To evaluate the interobserver agreement in histological and inflammatory parameter interpretation, Cohen’s kappa was applied.

### Statistical Analysis

Statistical analysis was performed using GraphPad-Prism 5 software (Graph Pad Software, San Diego, CA, USA) and MedCalc version 10.2.0.0 (MedCalc Software, Mariakerke, Belgium). A comparison of continuous variables was performed using the Mann–Whitney U-test (*t* test), as appropriate, while a comparison between categorical variables was performed through the use of chi-square statistics, using Fisher’s exact test. The inter-observer agreement of pathologists for the histological evaluation of morphological features of NPs and the type of inflammatory infiltrate was determined via Cohen’s kappa with confidence intervals (CIs). A *p* value < 0.05 was considered statistically significant [27].

## 3. Results

### Patients’ Characteristics and Histological and Inflammatory Features of the NPs

The sample analyzed included 143 patients divided into three main age-related subgroups: 6 in the pediatric age range (≤18 years old, mean 12.7), 58 adults (between 18 and 50 years old, mean 45.9), and 79 in the elderly age range (>50 years old, mean 70.3). The male-to-female ratio varied between the adult and elderly populations (3.8:1 and 3.4:1, respectively) and the pediatric population (1:1). The main clinicopathologic characteristics and histological features are reported in Table 1.

**Figure 1:**
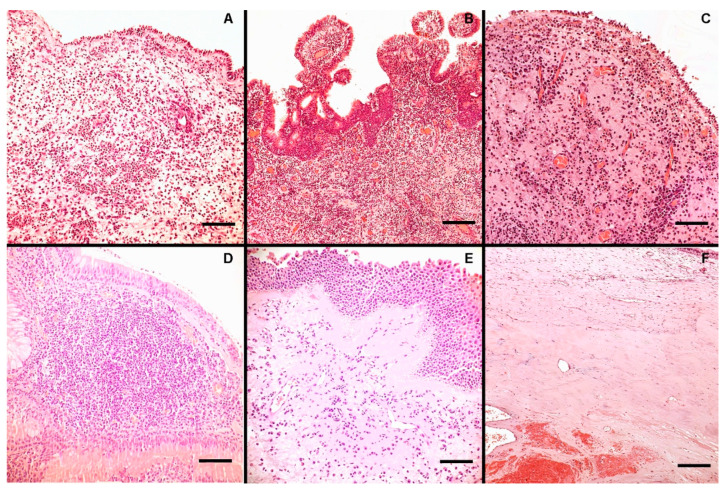
The figure shows some representative histological and inflammatory features in NPs: a rich eosinophilic infiltrate was noted in a young patient ((**A**), H&E, bar scale 200 µm), sometimes mixed with lymphocytes ((**B**), H&E, bar scale 400 µm). In the adult population, inflammation was mainly represented by lymphocytes and plasma cells ((**C**), H&E, bar scale 200 µm) that occasionally formed lymphoid nodules ((**D**), H&E, bar scale 200 µm). Squamous metaplasia was encountered in the superficial epithelium ((**E**), H&E, bar scale 200 µm). In the elderly population, inflammation was less evident, and cavernous-like structures with thrombotic stratification were seen ((**F**), H&E, bar scale 400 µm).

Generally, a noticeable myxoid stroma with vascular angectasias was seen in all NPs. A more pronounced inflammatory infiltration was seen mainly in the pediatric group, which was primarily distinguished by the presence of many eosinophils mixed with lymphocytes (Figure 1A,B). By contrast, in the adult population, inflammation was mainly represented by lymphocytes and plasma cells (Figure 1C), frequently with a perivascular distribution as well as the formation of subepithelial lymphoid nodules (Figure 1D). Squamous metaplasia was not rare in the superficial epithelium (Figure 1E). Moreover, adult patients showed a significantly higher neutrophil count compared to the elderly and pediatric populations. Last but not least, in the elderly population, inflammation was less evident, and eosinophils were only found in organized microcystic mucus collections; moreover, cavernous-like angecthatic structures with thrombotic stratification were seen (Figure 1F).

The mean count of lymphocytes (for 10 HPF) in the polyps was 493.9 (±SD 50.6) in adults, 266.7 (±SD 39.8) in the young subgroup, and 92.3 (±SD 30.0) in the elderly age group. Similar to those observed for lymphocytes, neutrophils and plasma cells had a higher count in adults (13.5 with a ±SD 4.3 and 20.8 with a ±SD 4.3, respectively), with a lower count in young patients (1.3 with a ±SD 1.0 and 11.0 with a ±SD 3.7, respectively) and in elderly patients (4.4 with a ±SD 1.7 and 11.0 with a ±SD 3.7, respectively). On the contrary, younger patients had a higher mean count of eosinophil cells (39.5, ±SD 9.9) in comparison to adult and elderly patients (8.26 with a ±SD 4.9 and 2.67 with a ±SD 0.8, respectively). Lymphoid nodules were mainly found in the NPs of the adult patients (33 out of the 58 patients, 56.9%), while in the elderly and young subgroups, 11 of the 79 and 1 out of the 6 patients (13.9% and 16.7%), respectively. Squamous metaplasia was prevalently observed in adult polyps (in 35 out of the 58 patients, 60.3%), while it was absent in young patients. Similarly, angecthatic vessels with thrombosis were absent in the young patients, prevalent in the elderly subgroup (44 out of the 79 patients, 55.7%), and less present in the adult subgroup (4 out of the 58 patients, 6.9%).

**Figure 2:**
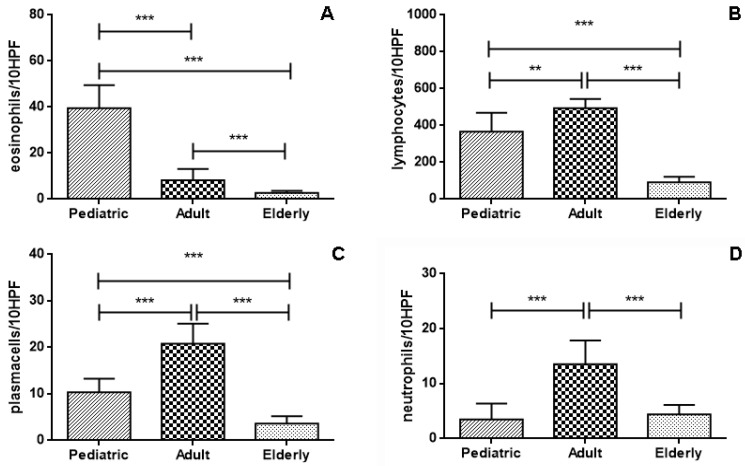
In the pediatric NPs subgroup, a significantly higher eosinophil count in comparison to both adult and elderly subgroups was noted ((**A**); *p* < 0.0001; Mann–Whitney *t* test). In the elderly subgroup, the lymphocyte count was significantly lower than in the adult and pediatric subgroups ((**B**); *p* < 0.0001; Mann–Whitney *t* test). Conversely, plasma cells and neutrophils were significantly higher in the adult NPs than in pediatric and elderly subgroups ((**C**,**D**); *p* < 0.0001; Mann–Whitney *t* test) (**: *p* < 0.001; ***: *p* < 0.0001).

**Figure 3:**
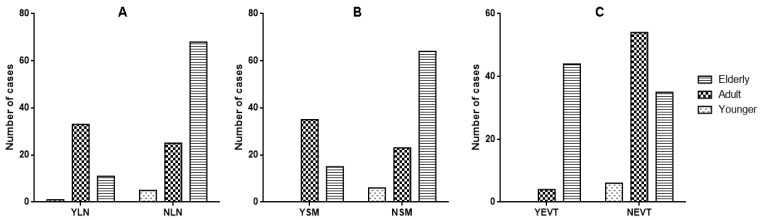
Age-related different distribution and statistical significance of lymphatic subepithelial nodules (YLN: presence, NLN: absence), squamous metaplasia (YSM: presence, NSM: absence) and ecthatic vessels with thrombosis (YEVT: presence, NEVT: absence). Lymphoid nodules were significantly higher in the adult subgroup in comparison to the elderly (*p* < 0.0001). (**A**). Similarly, squamous metaplasia was significantly present in the adult NPs with respect to the young (*p* = 0.0063) and elderly (*p* < 0.0001) (**B**). Lastly, there was a higher prevalence of angecthatic vessels with thrombosis in the elderly polyps than in the young (*p* = 0.010) and adult subgroups (*p* < 0.0001) (**C**).

In the young age subgroup, NPs had a significantly higher count of eosinophil cells in comparison to adult and elderly subgroups (*p* < 0.0001) (Figure 2A). Conversely, a higher count of lymphocytes was observed in adult NPs with respect to elderly (*p* < 0.0001) and pediatric ones (*p* = 0.0011), respectively (Figure 2B); similarly, a higher count of plasma cells was observed in adult NPs with respect to elderly (*p* < 0.0001) and pediatric ones (*p* < 0.0001) (Figure 2C). Neutrophils had a significantly lower count in elderly (*p* < 0.0001) and pediatric NPs (*p* < 0.0001) with respect to the adult subgroup (Figure 2D), while any significant difference between neutrophil counts in young and elderly NPs was encountered (*p* = 0.26) (Figure 2D). Regarding the presence of lymphoid nodules in NPs, we found that these were significantly higher in the adult subgroup in comparison to the elderly (*p* < 0.0001, OR 8.16, 95% CI from 3.586 to 18.57) (Figure 3A). Similarly, squamous metaplasia was significantly present in the adult NPs with respect to the young (*p* = 0.0063, OR 0.051, 95% CI from 0.003 to 0.948) and elderly (*p* < 0.0001, OR 6.49, 95% CI from 3.006 to 14.03) (Figure 3B). Lastly, we found a higher prevalence of angecthatic vessels with thrombosis in the elderly polyps than in the young (*p* = 0.010, OR 0.061, 95% CI from 0.003 to 1.127) and adult subgroups (*p* < 0.0001, OR 0.059, 95% CI from 0.019 to 0.178) (Figure 3C).

The interobserver reliability among pathologists for all analyzed parameters was in the range of almost perfect agreement, ranging from Cohen’s kappa 0.84 to 0.91.

## 4. Discussion

In the present study, we have analyzed the age-related morphological differences present in NPs, focusing on the qualitative features and characteristics of the inflammatory infiltrate. Using this approach, some evident statistically significant data have emerged between the three subgroups. In fact, in the pediatric group, many eosinophils intermingled with lymphocytes have been observed, while in the adult and elderly populations, inflammatory cells mainly constituted lymphocytes and plasma cells. Moreover, adult patients showed a significantly higher neutrophil count compared to the elderly and pediatric populations. Another intriguing feature is represented by squamous metaplasia in NPs, which is frequently encountered in the superficial epithelium of the adult subgroup compared to other groups. Finally, in the elderly population, inflammation was less evident, mainly seen in the eosinophilic component, but cavernous-like angecthatic structures with thrombotic stratification were commonly encountered. All of the above-mentioned data further support the idea that clinical work-up and treatment follow-up should be differently addressed in age relation to categories, taking into consideration the distinct histological features that have been reported.

To the best of our knowledge, little data have considered the histology of the inflammatory infiltrate in NPs, being only applied to eosinophils a standard value of tissue density required to define mucosal eosinophilia [1]. The value of 10 eosinophils/HPF or higher has been considered as a cut-off level by the European Position Paper on Rhinosinusitis and Nasal Polyps 2020 [1]. Nonetheless, in our cohort, NPs in children had a much higher tissue mean count of eosinophils (more than 30 eosinophils/HPF), while NPs in the other two age subgroups showed lower mean values. On the other side, no standard values have been reported in the literature regarding the average values of the other inflammatory elements in NPs. Therefore, our findings appear to be a starting point for further studies aimed at better characterizing age-related histological differences.

In order to explain the peculiar morphological appearance of NPs in the elderly population, in which more stromal and vascular changes with frequent thrombotic stratifications have been revealed, we retain that such finding could be related to a series of morpho-functional and immunologic modifications, commonly occurring in the sinonasal tract with aging. In fact, older CRSwNP patients are more likely to have higher tissue remodeling and more severe tissue edema [28]. Moreover, it has been shown that aging causes an increase in both nasal volume and intranasal resistance, both of which may attributed to the nasal tissues’ decreased suppleness [29,30,31]. In detail, the columella may retract, and the septal cartilage may break apart, and, due to a decrease in facial muscle mass and a weakening of the connective tissues, nasal tip support declines and the tip may become ptotic [32]. However, age dysregulates the function of the epithelial barrier in the sinonasal mucosa, with reduced mucociliary clearance, altered innate immunity, and an imbalance in the microbiome of this anatomical site [33]. As a consequence, older individuals become more susceptible to environmental factors, such as germs and allergens. On the other hand, it is well known that the levels of S100 family proteins, which are essential for both the maintenance and repair of the epithelial barrier [34], are noticeably decreased in older individuals [35]. Furthermore, reduced nasal blood flow in elderly people hinders the nares’ capacity to warm and humidify the air, which exacerbates nasal dryness [36]. These conditions produce thicker mucus compared to younger patients, which could, therefore, be more difficult to remove [37]. It should also be considered that mucosal excretory activity is further exacerbated by a reduction in sympathetic tone compared to parasympathetic activity [37]. Nonetheless, the overproduction of secretions may cause localized tissue hypoxia, which hinders muco-ciliary clearance even more [38]. Additionally, chronic hypoxia has been implicated in the pathogenesis of NPs; specifically, Shun et al. found that hypoxia may contribute to the formation of NPs by promoting autophagy in nasal polyp fibroblasts [39]. Moreover, Moon et al. documented the differentiation of hypoxia-stimulated myofibroblast in NPs through the activation of Nox4, a member of the NADPH oxidase family [40]. Finally, Lin et al. [41] demonstrated that hypoxia stimulates the production of vascular endothelial growth factor (VEGF), a key regulator of angiogenesis, in human nasal polyp fibroblasts, suggesting an explanation for the angiogenesis observed in NPs, such as that observed in our elderly subgroup.

It has been previously reported that in elderly patients, the pathogenesis of CRS, as well as NPs, is quite different, less linked with allergy and eosinophilic infiltration, and more with lower serum IgE levels, which is associated with weakened response to cytokines [35]. In fact, with aging, mucociliary clearance is reduced due to reductions in cilia beat frequency [42,43] and, as a result, the epithelium comes into longer contact with irritants. Additionally, it is well known that people over 50 years old seem to have a considerably reduced nasal cycle, which may further contribute to their sense of congestion [21]. Nonetheless, immunosenescence may exacerbate susceptibility to pathogens due to the fact that the immune system declines with age [37]. Furthermore, it has been underlined that the difference in CRS prevalence between elderly and young people showed that, after endoscopic sinus surgery, NPs recurred less often in the elderly, which is probably related to the lower eosinophilic infiltrate seen in this group [44]. Overall, anatomical and functional changes in the sinonasal regions during aging have been grouped under the term “presbynalis” [37], presumably explaining the different histological features observed in the NPs of different age groups.

Although the present study raises interesting considerations, some methodological biases have to be mentioned. Firstly, the retrospective analysis may justify the partial reliability of findings since we have had the opportunity to study only one sample per patient without any information about the potential relapse of NPs. Therefore, when considering future directions, this should be addressed to correlate the different age-related histological characteristics of the NPs with the risk of relapse. Secondly, the enrolled casuistry is not particularly large and comes from a single institution, and this may limit the generalizability of our results. Moreover, although we provided statistically significant results, the limited number of pediatric subjects affects the reliability of our findings and limits the scientific value and generalizability of our data. Therefore, further research on larger, multicenter cohorts with long-term histological evaluation of patients with NPs belonging to different age groups is needed to solve these limitations. Likewise, the high number of exclusion criteria compromises the possibility of providing statistically significant subgroup analyses, and the clinical picture of the population studied is not fully representative of usual patients with NPs, who usually have comorbidities such as asthma and allergic rhinitis. However, the aim of our study was to evaluate the histological characteristics of NPs in different age groups with the aim of reducing the number of confounding factors as much as possible, as demonstrated by the numerous exclusion criteria. A future direction could be reducing the exclusion criteria, which would be desirable to reflect the clinical reality of patients with NPs with their comorbidities and also allow for subgroup analysis. Lastly, a really intriguing perspective may be represented by a prospective study on NPs while trying to correlate the presence of the histological specificities of NPs at different ages of life with recently introduced biological drugs, such as monoclonal antibodies, and the treatment failure characterized by subsequent clinical relapse. In fact, biologics have been reported as a recent tool in the treatment of uncontrolled severe CRSwNP patients who have not had a positive response to traditional medications or surgery [45,46,47]. However, the effectiveness of biologics firmly relies on understanding the age-related pathophysiology of CRSwNPS since the precise selection of patients for targeted treatments represents the fundamental requirement for therapeutic success and efficient means of treating comorbid conditions like severe asthma.

## Figures and Tables

**Table 1:** Clinicopathological data of patients and histopathological features in nasal polyps (NPs).

Variable		Total		Younger	Adult	Elderly
*Number of patients, n (*%*)*		143 (100)	6 (4.2)	58 (40.6)		79 (55.2)
*Age, mean (±SD)*	51.7 (15.8)	12.7 (4.5)	45.9 (9.2)	70.3 (7.5)		
*Male gender, n (*%*)*		110 (76.9)	3 (50)	46 (79.3)		61 (77.2)
*Female gender, n (*%*)*		33 (23.1)		3 (50)	12 (20.7)	18 (22.8)
*Eosinophils/10HPF, mean (*±*SD)*	6.5 (8.3)	39.5 (9.9)	8.26 (4.9)	2.67 (0.8)		
*Lymphocytes/10HPF, mean (*±*SD)*	262.5 (198.3)	266.7 (39.8)	493.9 (50.6)	92.3 (30.0)		
*Neutrophils/10HPF, mean (*±*SD)*	8 (5.5)	1.3 (1.0)	13.5 (4.3)	4.4 (1.7)		
*Plasma cells/10HPF, mean (*±*SD)*	10.9 (8.9)	11.0 (3.7)	20.8 (4.3)	3.6 (1.6)		
*Lymphoid nodules, n (Y*/*N)*	45/98	1/5	33/25	11/68		
*Squamous metaplasia, n (Y*/*N)*	50/93	0/6	35/23	15/64		
*Angioectasias, n (Y*/*N)*	48/95	0/6	4/54	44/35		

SD: standard deviation; HPF: high power field; NPs: nasal polyps; Y: presence; N: absence.

## Data Availability

The datasets used and/or analyzed during the current study are available from the corresponding author upon reasonable request.
